# A Route to Terahertz Metamaterial Biosensor Integrated with Microfluidics for Liver Cancer Biomarker Testing in Early Stage

**DOI:** 10.1038/s41598-017-16762-y

**Published:** 2017-11-27

**Authors:** Zhaoxin Geng, Xiong Zhang, Zhiyuan Fan, Xiaoqing Lv, Hongda Chen

**Affiliations:** 1 School of Information Engineering, Minzu University of China, Beijing, China; 2 State Key Laboratory of Integrated Optoelectronics, Institute of Semiconductors, Chinese Academy of Sciences, Beijing, China

## Abstract

Engineered Terahertz (THz) metamaterials presented an unique characteristics for biosensing application due to their accurately tunable resonance frequency, which is in accord with vibrational frequency of some important biomolecules such as cancer biomarker. However, water absorption in THz regime is an obstacle to extend application in trace biomolecules of cancer antibody or antigen. Here, to overcome water absorption and enhance the THz biosensing sensitivity, two kinds of THz metamaterials biosensor integrated with microfluidics were fabricated and used to detect the Alpha fetoprotein (AFP) and Glutamine transferase isozymes II (GGT-II) of liver cancer biomarker in early stage. There were about 19 GHz resonance shift (5 mu/ml) and 14.2 GHz resonance shift (0.02524 μg/ml) for GGT-II and AFP with a two-gap-metamaterial, respectively, which agreed with simulation results. Those results demonstrated the power and usefulness of metamaterial-assisted THz spectroscopy in trace cancer biomarker molecular detection for biological and chemical sensing. Moreover, for a particular cancer biomarker, the sensitivity could be further improved by optimizing the metamaterial structure and decreasing the permittivity of the substrate. This method might be powerful and potential for special recognition of cancer molecules in the early stage.

## Introduction

A lots of in-depth research results and clinical diagnose show that cancer cells have different molecular subtypes whose characteristics play an important role in biological behavior of cancer. If only from the level of tissue cells to diagnose the patients with a cancer, maybe the result is that the patients would be in the least state of the cancer. Therefore, the detection of cancer should be from molecule level in early stage caner, which could help the patient perform early detection and early treatment. However, there are also the difficulties faced by existing diagnosis methods^1–6^. For early cancer patients, there are trace amount cancer biomarkers in the serum, such as enzymes, cytokines, specific proteins, which could indirectly reflect the existing of tumor cells. Therefore, variety of biosensors were developed and used to sensitively detect these biomarkers, which are very important for early detection of cancer. Especially, optical biosensors, which are non-destructive, high sensitive and rapid-detecting techniques, have aroused a great of research interesting.

Although there are many optical methods for biomarker sensing detection^7–16^, such as fluorescence-based microbial detection, surface plasmon resonance (SPR)^7^, localized surface plasmon resonance (LSPR)^8,15,16^, surface enhanced Raman scattering (SERS)^9,11–14^, and terahertz (THz)^4,5,17–19^. Because of that the existing sensing detection technologies maybe have drawbacks in sensitivity, detection limit, volume of sample, cost, vibrational frequency of biomarker and water absorption, those methods are unable to meet the requirement of early diagnosis of cancer. Therefore, novel techniques or new materials for high sensitivity detection of cancer biomarkers in early stage are needed increasingly.

Recently, THz spectroscopy technology based on metamaterials have become a promising biomarker detection method. On one hand, frequency range of THz wave (0.1 THz to 10 THz) is in accord with vibrational frequencies of some important biomolecules (proteins, RNA, and DNA)^1^, which make it is possible to detect the vibration of biomolecules. Besides, THz spectroscopy technology has some other merits such as label-free, non-contact, and non-destructive inspection on target biomolecules. All of these merits imply that THz spectroscopy technology is suitable to  detect biomolecules. On the other hand, with the development of the micro-nanofabrication technology, novel THz metamaterials were designed to obtain a tailored electromagnetic response, which are very sensitive to micro-environment medium change on the surface of the metamaterials. Especially, to get sharp transmission spectra in bio-sensing, metamaterials often were designed as asymmetric structures, which easily cause Fano resonances if the structure is reasonable and the substrate effect is eliminated^20^. The spectra shape of Fano resonances is asymmetric and sharp, which could offer high quality factor. Therefore, the Fano resonance-based metamaterials are a promising materials to detect biomolecules with extremely low concentration.

However, THz sensor have typically been limited to dry or partially hydrated specimen due to strong water absorption at THz frequencies, which mean that how to reduce the liquid tested sample is an important direction for extending THz biosensor application. Microfluidic chip maybe presents a new method to overcome water absorption because microfluidic chip offers many advantages such as very small volume of samples, low cost, rapid analyzing and so on^21^. Due to these wonderful properties, microfluidic chip could be introduced into THz biosensors as a promising assistant^4,22–25^. Microfluidics could avoid this drawback with its little usage of liquid, tight fluidic confinement and precise fluidic control at micro-scale. If the detected biomolecules (proteins, RNA, DNA and so on) are dissolved in liquid sample through microfluidic technology^1,26–29^, THz sensor integrated with microfluidic chip are applied extensively because strong water absorption at THz frequencies can be overcome. Therefore, the THz metamaterials combined with microfluidics could be favorable for sensing in aqueous environment because the ultra-thin water layer can not suffer from the significant loss in the THz wave transmission.

Herein, we developed a novel THz metamaterials biosensor integrated with microfluidics, which was used to detect the biomarker of the liver cancer in early stage. In particular, the THz metamaterials biosensor was functionalized with the antibody specific to antigen of the cancer biomarker in aqueous environment by microfluidics system, which could enable the specificity detection for liver cancer biomarkers. Before the THz test, the microfluidics layer, which was made of poly (dimethylsiloxane) (PDMS), would be peeled off from the metamaterials to reduce the loss of THz wave transmission. The residual water layer would also be blow-dried by nitrogen gas. The resonance frequency shift in THz metamaterials along with the captured biomolecules was recorded and investigated in detail as a function of the dielectric constants and the concentration of liver cancer biomarker which was in accordance of the dielectric constant measurement of individual antigen of liver cancer.

## Results and Discussion

### Design of metamaterials biosensor chip

**Figure 1:**
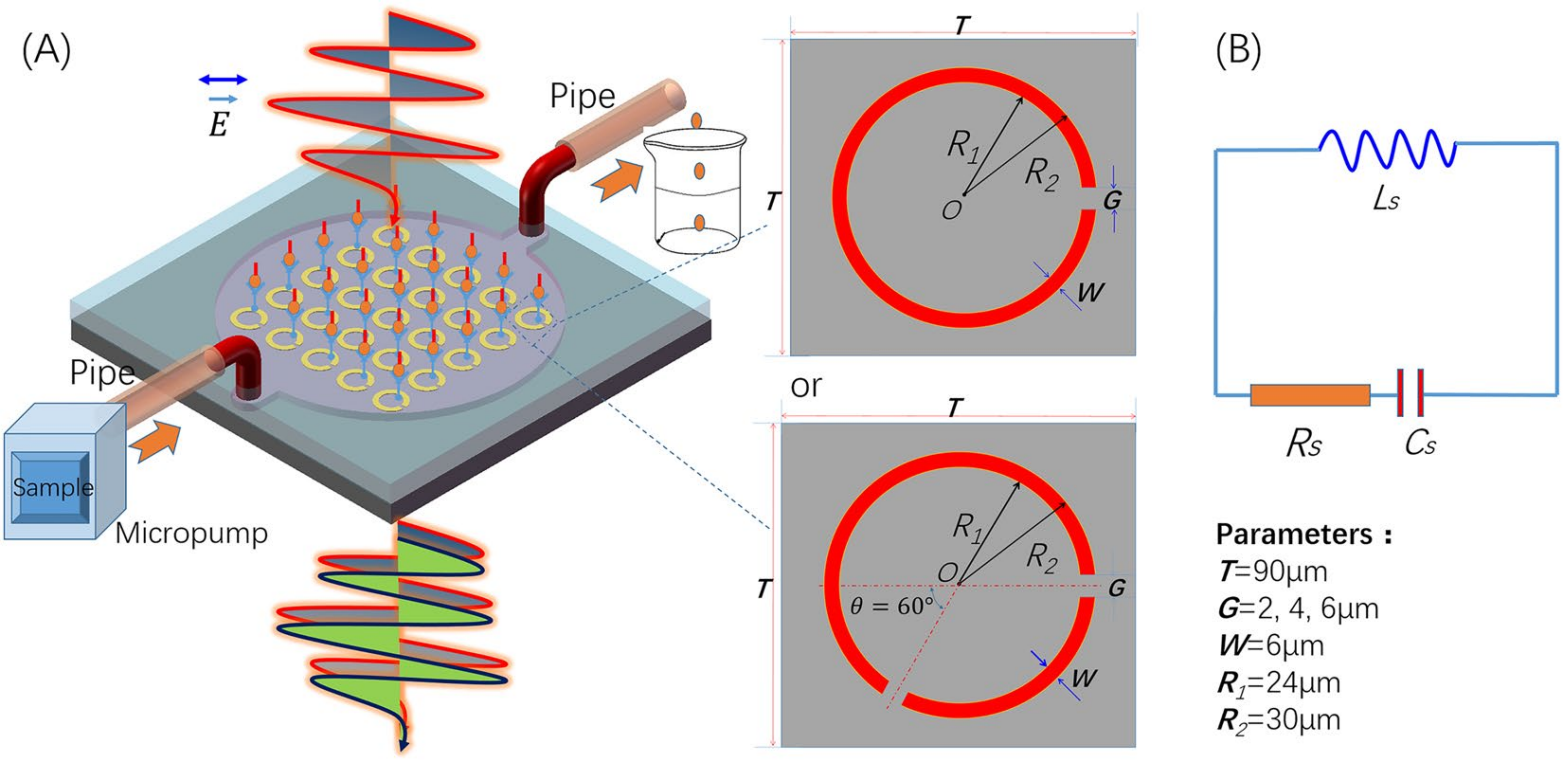
(**A**) The sketch of THz metamaterials biosensor chip integrated with microfluidics; (**B**) Equivalent circuit with {RLC}s for the SRRs.

Figure 1(A) depicts the diagram of a biosensor based on THz metamaterials chip integrated with microfluidics. The main component of the proposed THz biosensor consists of metal split-ring resonators (SRRs) and PDMS microchannel. The SRRs are made of metal loop with one or two square gaps. The THz wave perpendicularly launch into the THz chip integrated with microfluidics. The incoming THz wave with electric field polarized in the direction parallel to the SRRs axis excites the fundamental inductive-capacitive resonance of the SRRs, therefore, the inducing current appear in the nearby metal SRRs. The electric energy and magnetic energy transfer between inductance and capacitance. The strong electric field is established across the dielectric gap when fundamental resonance happens. The THz metamaterials biosensor chip could be approximated by fundamental circuit elements, as shown in Fig. 1(B). The SRRs round loop is regarded as the inductance *L* and the gap for the capacitance *C* based on fundamental circuit theory^6^.

To achieve a higher sensitivity of detecting the cancer biomarkers in early stage, the THz metamaterials biosensor needs a sharp edge in its frequency response and a point of high concentration of electric field to enable the detection of small changes in the dielectric environment. THz propagation could be accomplished by quasi-optical techniques using transmission of THz free-space radiation. Quasi-optical THz-systems are widespread since they offer a simple method for low-loss and broad-band THz transmission. However, these measurement systems are rather bulky because large collimators with focal lengths of several centimeters are used. Additionally, water vapor should be excluded from the THz beam path to eliminate the influence of water in the transmitted THz signal spectrum.

To overcome the water absorption, microfluidics technology was introduced in THz metamaterials-based biosensor chip because the microfluidics enable only the minute quantity of the sample inside a microchannel by utilizing the microelectromechanical system (MEMS) technology. Meanwhile, the testing box was filled with Nitrogen.

Metamaterial was fabricated on a high-resistivity silicon substrate. The width (*W*) of the split ring was 6 µm. The inner radius *R*_1_ and outer radius *R*_2_ were 24 µm and 30 µm, respectively. The period of split-ring resonators (SRRs) was 90 µm. The size of gap (*G*) changed from 2 µm to 6 µm. This sensing mechanism is that the equivalent capacitance of the SRRs is sensitive to the change of environment refractive index. When the biomarker of cancer is modified on the SRRs-MM structure, the equivalent capacitance is changed, in turn, the resonant frequency shift which indicates the presence of biomarker of cancer.

Liver cancer antibody Alpha fetoprotein (AFP) (1 μg/mL solution in PBS buffer) were coupled to the surface of the SRRs through chemical reaction between amidogen beside the alkaline aminophenol (Arg and Lys) of IgG and the active carboxyl; and maintaining 16 hours at 4 °C. A monolayer of antibody was formed on the Au SRRs. The supernatant of Mouse anti-AFP was thoroughly rinsed with 0.01 M PBS buffer more than 10 times and then dried by N_2_ blowing with low-pressure; Redundant active ester groups were enclosed by 1 M ethanolamine aqueous solution, and maintaining 120 min. An alternate experimental approach was that a bovine serum albumin (BSA) solution (0.1 mg/mL) was used to block the nonspecific binding sites of antibody-modified SRRs; The Phosphate-buffered saline (PBS) with 0.01 M was pumped into the microchamber to rinse out redundant ethanolamine aqueous solution and then dried by N_2_ blowing with low-pressure.

AFP antigen or Glutamine transferase isozymes II (GGT-II) antigen (Tianjin Tumour Hospital) with different concentrations was injected into the chamber to incubate for more than 40 min. The reflection spectra or transmission spectra were recorded through THz-TDS setup during different time.

### Modeling of metamaterials biosensor response

**Figure 2:**
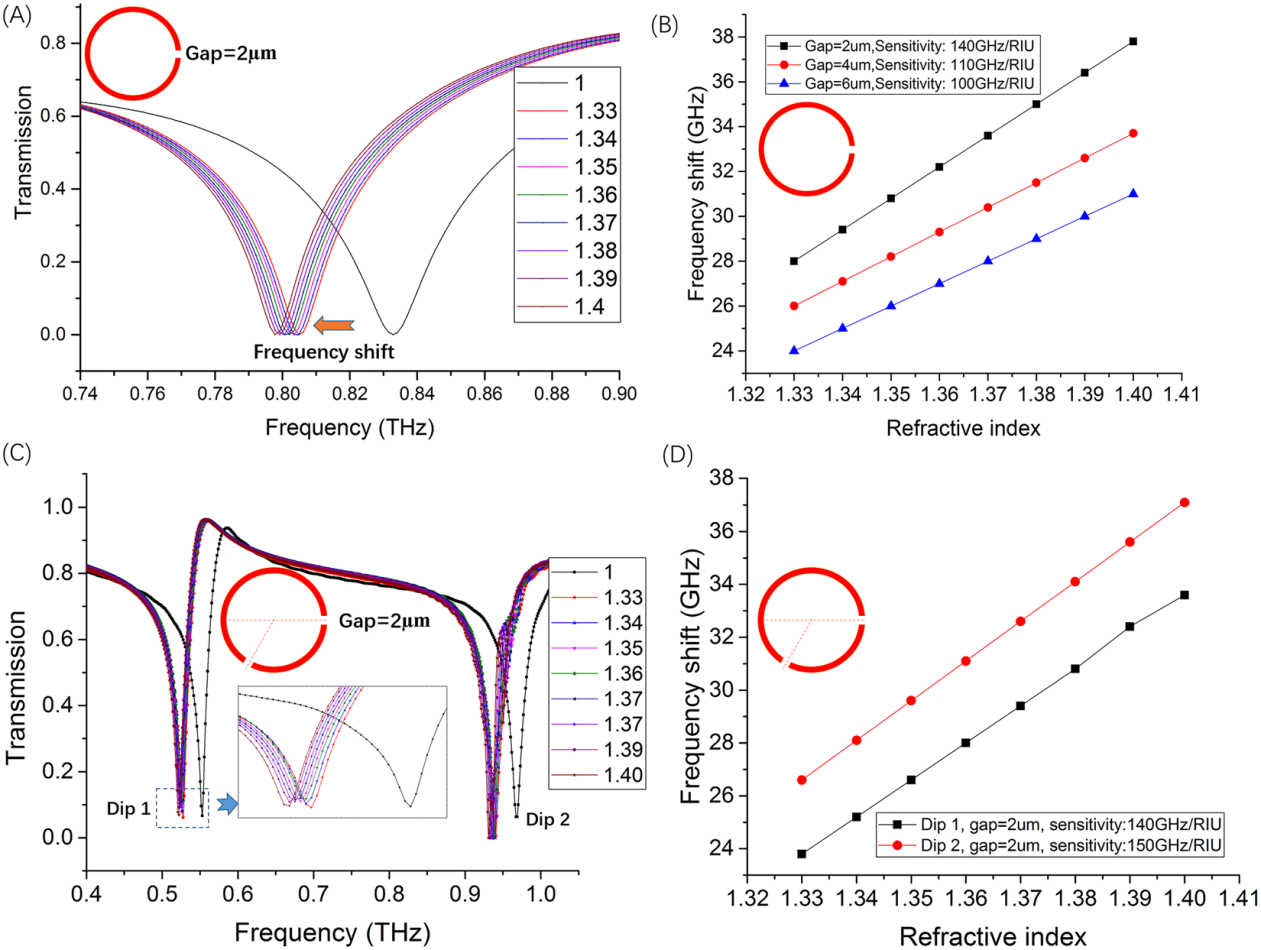
The simulated results which could guide to design the structure of THz metamaterials biosensor chip. (**A**) Transmission spectra of SRRs with a 2 µm gap under different refractive index; (**B**) Sensitivity of SRRs with 2 µm, 4 µm or 6 µm gap; (**C**) Transmission spectra of SRRs with two gaps (2 µm) under different refractive index; (**D**); Sensitivity of SRRs with two 2 µm gaps for Dip 1 and Dip 2.

To explore the characteristics of the THz metamaterials biosensor integrated microfluidics and to guide design of the chip, the Finite-Difference Time-Domain (FDTD) solution software was applied to explore the THz biosensing sensitivity. Two kinds of SRRs were swept by changing the gap width (2 µm, 4 µm and 6 µm) of the SRRs. Typical transmission spectra of SRRs with one gap (2 µm) under different ambient refractive index shown in Fig. 2(A). The results shown that the resonance absorption frequencies decrease when the ambient refractive index increase. It was clear that any change in the liquid permittivity causes a transmission curve parallel shift to low frequency. Meanwhile, the Q factor increase when the liquid permittivity or the ambient refractive index increase. The results of the SRRs with gaps (4 µm and 6 µm) demonstrated the same tendency. The relationship between the frequency shift and the ambient refractive index was showed in Fig. 2(B). The sensitivities of SRRs, which equaled to the slope of the lines in Fig. 2(B), reached 140 GHz/RIU, 110 GHz/RIU and 100 GHz/RIU when the width of gap was 2 µm, 4 µm or 6 µm, respectively. It illustrated that the sensitivity increased when the gap decreased. The reason lies in that the small gap could create the hot-spot of plasmonic resonance^23^.

To enhance the sensitivity of SRRs and Q value, asymmetric structure of SRRs was used to produce the Fano resonance. The representative Fano resonance of SRRs with 2 gaps under different ambient refractive index shown in Fig. 2(C). The central angle formed by two radius cross the center of two gap was 120°. The transmission spectra of this asymmetric structure SRRs had 2 dips and with high Q value, which had the plasmonic resonant characteristics. The two dips presented a redshift tendency with the ambient refractive index increasing; however, the value of the redshift was different. The larger redshift happened in the higher frequency. The reason was that the resonant characteristic could be resonantly enhanced to the bonded inter- or intra-molecular of sample in THz region, resulting in a much larger redshift. The sensitivity was 140 GHz/RIU and 150 GHz/RIU at dip 1 and dip 2, respectively (Fig. 2(D)).

Compared the simulation results for one gap with different gap sizes (2 µm, 4 µm or 6 µm) and in Fig. 2(C, D) for two gaps with 2 µm, it illustrated that the resonance shift decreased and the frequency shift increased with the ambient refractive index increasing. Meanwhile, the ambient refractive index was a function of the permittivity of the tested sample. To analysis that how the permittivity around the SRRs affected the characters of THz-metamaterial biosensor chip was a fundamental and important step, which was a key factor to understand THz-metamaterials sensing.

Based on fundamental circuit theory, when the THz wave perpendicularly illuminated on the SRRs surface, the whole resonance circuit formed with gold SRRs shown resonance phenomenon and the transmitted spectrum had a sharp dip at resonance frequency. The mutual inductance (M) among SRRs was omitted, because the distance between the SRRs was more than 30 μm. Therefore, the impedance (*Z*) of the SRRs resonator could be presented as

1 $$Z=R+j\omega L+1/j\omega C$$

where, *R*, *C* and *L* are the total resonance resistance, capacitance and inductance at resonance frequency. The capacitance *C* is a function of the permittivity around the gap, and can be roughly approximated by *C* = *C*_0_ + *ε*_*sam*_ *C*_*C*_, where *C*_0_ includes the capacitive effect from the dielectric substrate, microchannel walls, and surrounding space, excluding the channel cavity. *Cc* the capacitance of microchamber filled with air. If the microchamber is filled with different testing samples, *C*_*c*_ is described as ε_sam_ *C*_*c*_. Meanwhile, it is assumed that the fringing field pattern effect is neglected with sample loading. The total resonance resistance *R* and resonance capacitance *C* of the SRRs integrated with microfluidics are a function of the sample permittivity (ε_sam_). This means that *R* and *C* change with injected different testing sample. Therefore, the resonance characteristics of SRRs would be affected by the parameter of tested sample, such as ε_sam_. The resonance frequency (ω) is rewrited as

2 $$\omega =1/\sqrt{LC}=1/\sqrt{L({C}_{0}+{\varepsilon }_{sam}{C}_{C})}$$

Therefore, from *Eq*. (2), the resonance frequency is dominated by the complex permittivity of the testing sample. By recording the transmission resonance frequency, the dielectric characteristics of the liquid sample would be retrieved. Meanwhile, permittivity *ε*_*sub*_ of the substrate also is a crucial factor for the character of SRRs. Especially, in the microfluidic environment with permittivity *ε*_*sam*_ of target sample, the resonance frequency is approximate function of effect permittivity *ε*_*eff *_(*ε*_*eff*_ = *ε*_*sub*_ + *ε*_*sam*_). From this equation, the effect permittivity increases in the microfluidic environment, therefore, the resonance frequency would decrease. Usually, for planar metamaterials THz wave biosensor, the sensitivity is the shift of the resonance frequency caused by the refractive index change or transmitted intensity variation. The THz dip frequency shift is approximately linear with changes in refractive index of the surrounding medium. To analysis the sensitivity to the THz metamaterials biosensor chip based on SRRs integrated with microfluidics, the frequency shift of the resonance dip with per-refractive index unit (GHz/RIU) should be considered (Fig. 2 (B) and (C)). However, refractive index is function of permittivity. Therefore, the permittivity *ε*_*sam*_ of testing sample and the permittivity *ε*_*sub*_ of the substrate play the key role in the sensitivity of the chip. Especially, the changes among permittivity of different testing sample are related with the sensitivity. Therefore, the ratio between *ε*_*sam*_ and *ε*_*eff*_ show as

3 $$\frac{{\varepsilon }_{sam}}{{\varepsilon }_{eff}}=\frac{{\varepsilon }_{sam}}{{\varepsilon }_{sub}+{\varepsilon }_{sam}}=\frac{1}{\frac{{\varepsilon }_{sub}}{{\varepsilon }_{sam}}+1}$$

From the *Eq*. (3), $\frac{{\varepsilon }_{sub}}{{\varepsilon }_{sam}}$ is a key factor for sensitivity. Meanwhile, it shows that sensitivity increase if the permittivity *ε*_*sub*_ decrese. The substrate with high permittivity will have a much larger base capacitance than the thin film substrate with low permittivity as the electric field penetrates deeply in the substrate at resonance, which decreases the resulting magnitude of the resonance shift due to the overlaid analyte. There are two kinds of methods to improve the sensitivity: 1) To find low permittivity *ε*_*sub*_ material with low absorption in THz regime; 2) To test high permittivity *ε*_*sam*_ sample. However, the tested target often is solution. Water absorption in THz regime maybe affect the sensitivity. Therefore, the microfluidics is applied in the THz metamaterials biosensor to overcome the water absorption in THz regime. In this paper, for liquid micro-sample, the refractive index sensitivity *S* of SRRs integrated with microfluidics chip is defined dip frequency shift per-refractive index unit. Sensitivity could be characterized as

4 $$S={\rm{\partial }}f/{\rm{\partial }}n\propto {\rm{\Delta }}f/{\rm{\Delta }}{\varepsilon }_{sam}$$

where, *S* is sensitivity, whose unit is GHz/RIU (RIU=Refractive Index Unit), ${\rm{\partial }}n$ represents the change of the refractive index; ${\rm{\partial }}f$ represents change of resonance frequency; ${\rm{\Delta }}{\varepsilon }_{sam}$ represents change of sample permittivity in different testing steps.

**Figure 3:**
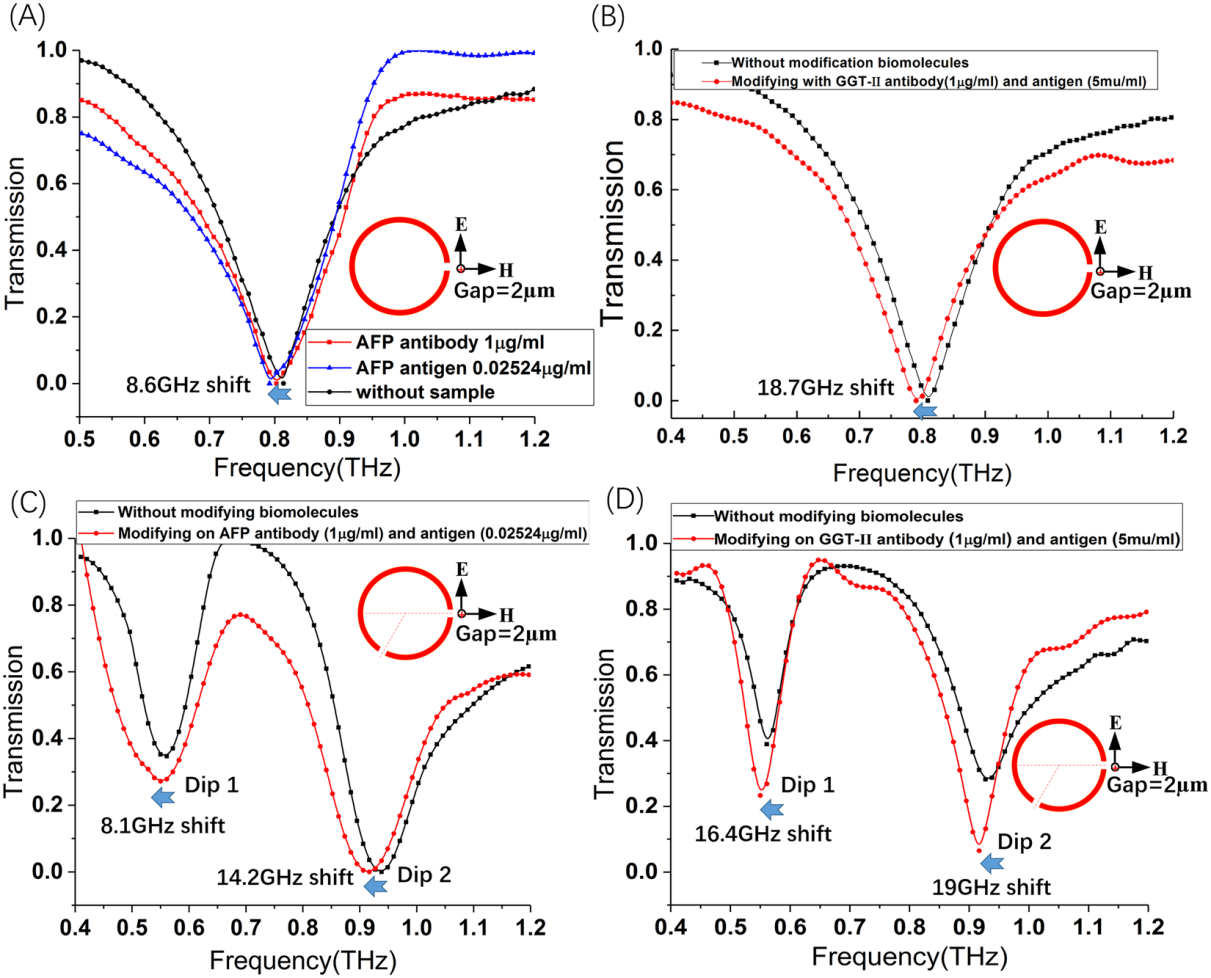
The typical tested results of liver cancer biomarker. (**A**) The results for AFP of SRRs with a 2 μm gap; (**B**) The results for GGT-II liver cancer marker testing of SRRS with a 2 μm gap; (**C**) The results for AFP of SRRs with 2 gaps (2 μm); (**D**) The results for GGT-II liver cancer marker testing of SRRS with 2 gaps (2 μm).

Because SRRs bio-sensing integrated with microfluidics is based on THz spectral dip frequency shifts, the precision of sensitivity can be achieved with respect to changes in the refractive index depends on the sensitivity, *S*, and the peak line width. The period and morphology of SRRs affect the peak line width, which have an effect on the frequency shift. Especially, asymmetrical structure would produce the Fano resonance with sharp dip in transmission spectrum. A figure of merit (FOM) obtained by dividing the sensitivity by the resonance frequency line width is widely used to characterize SRRs sensing capabilities. FOM is described as:

5 $$FOM=S/FWHM$$

where, FWHM repents full width at half maximum. Seen from Fig. 2(A) and (C), for spectra biosensor chip, the value of FOM is bigger, the performance of the biosensor is better. The sensing tendency of THz-metamaterials biosensor in simulation results is consist with that of the tested results (Fig. 3).

In fact, AFP is a kind of special protein in the blood, which is at very low level in serum of normal adult. And in serum of patients with liver cell carcinoma, the concentration of AFP increases significantly. Therefore, AFP is used as a tumor marker to help detecting and diagnosing cancers of the liver, testicles, and ovaries. The test is often ordered to monitor people with chronic liver diseases, such as cirrhosis, chronic hepatitis B, because they have an increased lifetime risk of developing liver cancer. A healthcare practitioner may order an AFP test, along with imaging studies, to try to detect liver cancer when it is in the earliest and the most treatable stages. Meanwhile, GGT-II is considered to be the best liver cancer markers besides AFP. Currently, GGT-II is often regarded as a carcinoma embryo protein produced by liver cell. Because the GGT-II sugar chain structure would change in the early stage of the liver cancer, GGT-II could play an important role for liver cancer diagnosing in early stage. However, AFP and GGT-II are extra small in the early stage of liver cancer, to detect the trace biomarker of liver cancer is important. However, there are many kinds of the biosensor technology, which are used to detect the biomarker of the cancer. The concentration of the biomarker is higher than the standard of the clinical testing results.

Based on theory and simulation results, two kinds of the SRRs (one with a gap (2 µm) and another with two gaps (2 µm)) were used to detect cancer biomarker such as AFP and GGT-II. To certify and extend the methods, the standard processes were used to clinical early liver cancer testing. The processes of SRRs substrate pre-functionalization were almost the same to the immunoreaction between AFP antibody and AFP antigen of liver cancer. The transmission spectra of the liver cancer antibody AFP (1 µg/ml) and the blood serum antigen (0.02524 µg/ml) of liver cancer were tested by using the SRRs biosensor with a 2 µm gap. The frequency shift of transmission spectra was 8.6 GHz before and after injecting AFP antibody and blood serum antigen (shown in Fig. 3(A)). Another liver cancer biomarker GGT-II antibody (1 µg/ml) and GGT-II antigen (5 mu/ml) were also tested by same way. There was a frequency shift of transmission spectra 18.7 GHz before and after injecting GGT-II antibody and antigen (Fig. 3(B)). The experiment results demonstrated that THz metamaterials biosensor chip integrated with microfluidics for THz spectroscopy of biomolecules (liver cancer biomarker) was an important method for cancer early diagnosis. Performing spectroscopy of biomolecules (liver cancer biomarker) in THz metamaterials biosensor integrated with microfluidic chip could be advantageous for the following reasons: 1) Microfluidic chip could easily be integrated with SRRs components to realize multifunctional spectroscopy platforms. 2) The small volume of microfluidic chip could facilitate the use of low-power THz-TDS systems, which could avoid excess water absorption and enable the spectroscopy of pico-mole quantities of biomolecules.

A representative result on the highly sensitive detection of low-density molds by using the THz metamaterials was demonstrated in the following. Figure 3(C) and (D) shown two dips of transmission spectra of the SRRs with two gaps (2 μm), which was the results of testing AFP and GGT-II. The transmission dips shifted 8.1 GHz and 14.2 GHz with the concentration of the AFP antibody 1 μg/ml and AFP antigen 0.02524 μg /ml which have full-width at half maximum (FWHM) of about 0.15 THz (left) and 0.2 THz (right) in Fig. 3(C), respectively. There are almost the same results shows in Fig. 3(D) for GGT-II with the concentration GGT-II antibody (1 μg/ml) and GGT-II antigen (5 mu/ml), respectively.

**Figure 4:**
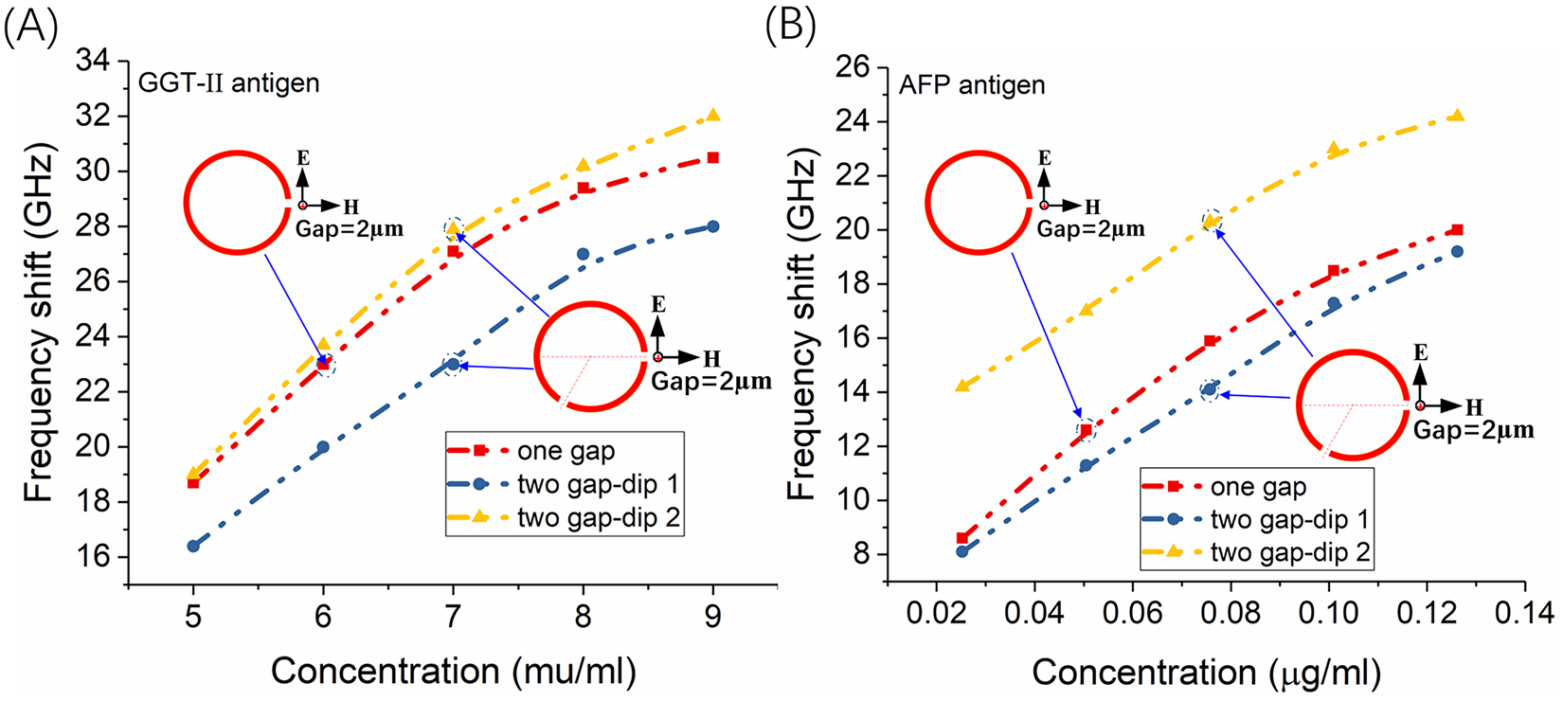
The spectra frequency shift for liver cancer biomarker in different concentration. (**A**) The frequency shift with different concentration of GGT-II antigen and fixed concentration GGT-II antibody 1 μg/ml. (**B**) The frequency shift with different concentration of AFP antigen and fixed concentration AFP antibody 1 μg/ml.

The dose-dependent manner was explored under fixed concentration of antibody at 1 μg/ml. For example, to investigate the frequency shift with different concentration GGT-II antigen (from 5 mu/ml to 9 mu/ml), the GGT-II was fixed at 1 μg/ml and the results shown in Fig. 4(A). The results illustrated that the frequency shifts are nearly linear growth. The same manner (shown Fig. 4(B)) for different concentration AFP antigen (from 0.02524 μg /ml to 0.1262 μg /ml). The value 0.02524 μg /ml for AFP antigen and 5mu/ml for GGT-II antigen are smaller the clinical standard, which mean that this kind of THz biosensor integrated with microfluidics could be used to detect the liver cancer biomarker.

## Conclusion

Two kinds of the SRRs have been designed, fabricated, and characterized on bulk silicon substrates, which integrated with microfluidics for bio-sensing applications. The testing results of two kinds of liver cancer biomarker AFP and GGT-II show agreement between experiment and simulation. From the results, we also found a linear relationship of normalized frequency shift with the reciprocal of refractive index of the substrate. Moreover, the sensitivity could be further optimized by metamaterial design for a particular biomolecule as necessary. This emergent field offers another promising platform for sensing with the characteristics of designable and tunable resonant features. As a young research field, more real bio-systems and applications are desirable and would be developed. This method might be very helpful for desirable bio-recognition in biology, medicine, and drug industry.

## Materials and methods

### Materials for THz bio-sensing chip fabrication

Glycol, ethanol and glycerol (Sinopharm Chemical Reagent Beijing, Co. Ltd) were used as MOS (Metal-oxide-semiconductor) level. Water was triply distilled. Polydimethylsiloxane (PDMS Sylgard 184, Dow Corning Corporation) pre-polymer was combined with amounts of cross-linker (wt. ratio 10:1). The SU-8 (type: 2075) was purchased from Microchem.

### Materials for sensing testing

1-ethyl-3-(3-dimethylamino-propyl) carbodiimide-HCI (EDC), sulfo-nhydroxysuccinimide (S-NHS), ethanolamine, NaCl, dithiodiglycolic acid were purchased from Sigma; Mouse Anti-AFP, phosphate buffer solution (PBS, 0.01 M, pH 7.4) were purchased from Beijing Biosynthesis Biotechnology Co. Ltd; The buffer used in the experiments was prepared using double glass-distilled water. AFP-antibody and Glutamine transferase isozymes II (GGT-II) for liver cancer and antigen in the serum of liver cancer patients was offered by Tianjin Medical University.

### TDS spectroscopy

A standard a THz-TDS setup based on photoconductive switches are used to characterize the THz transmission spectra of the metamaterials with cancer biomarkers (antibody and antigen). Terahertz radiation is generated and detected using photoconductive switches driven by 10 mW optical pulse trains from an 800 nm, 80 fs, 100 MHz mode locked sapphire femtosecond laser. The emitted terahertz radiation is collimated by a high-resistivity silicon lens and parabolic mirrors. The samples were measured at ambient temperature in a dry nitrogen atmosphere at 25 °C.

### Metamaterials fabrication

**Figure 5:**
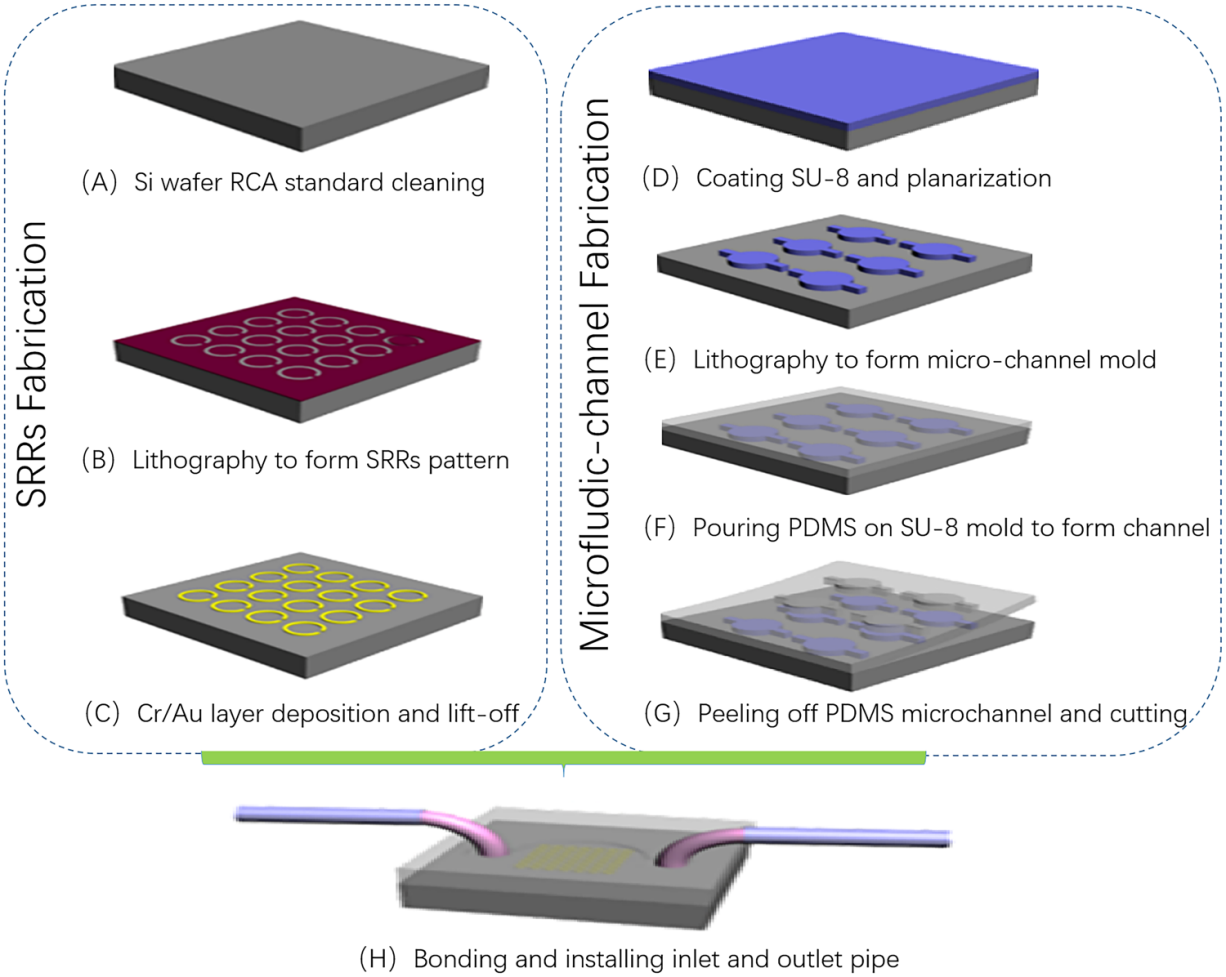
Fabrication processes of the THz metamaterials biosensor chip integrated with microfluidics.

**Figure 6:**
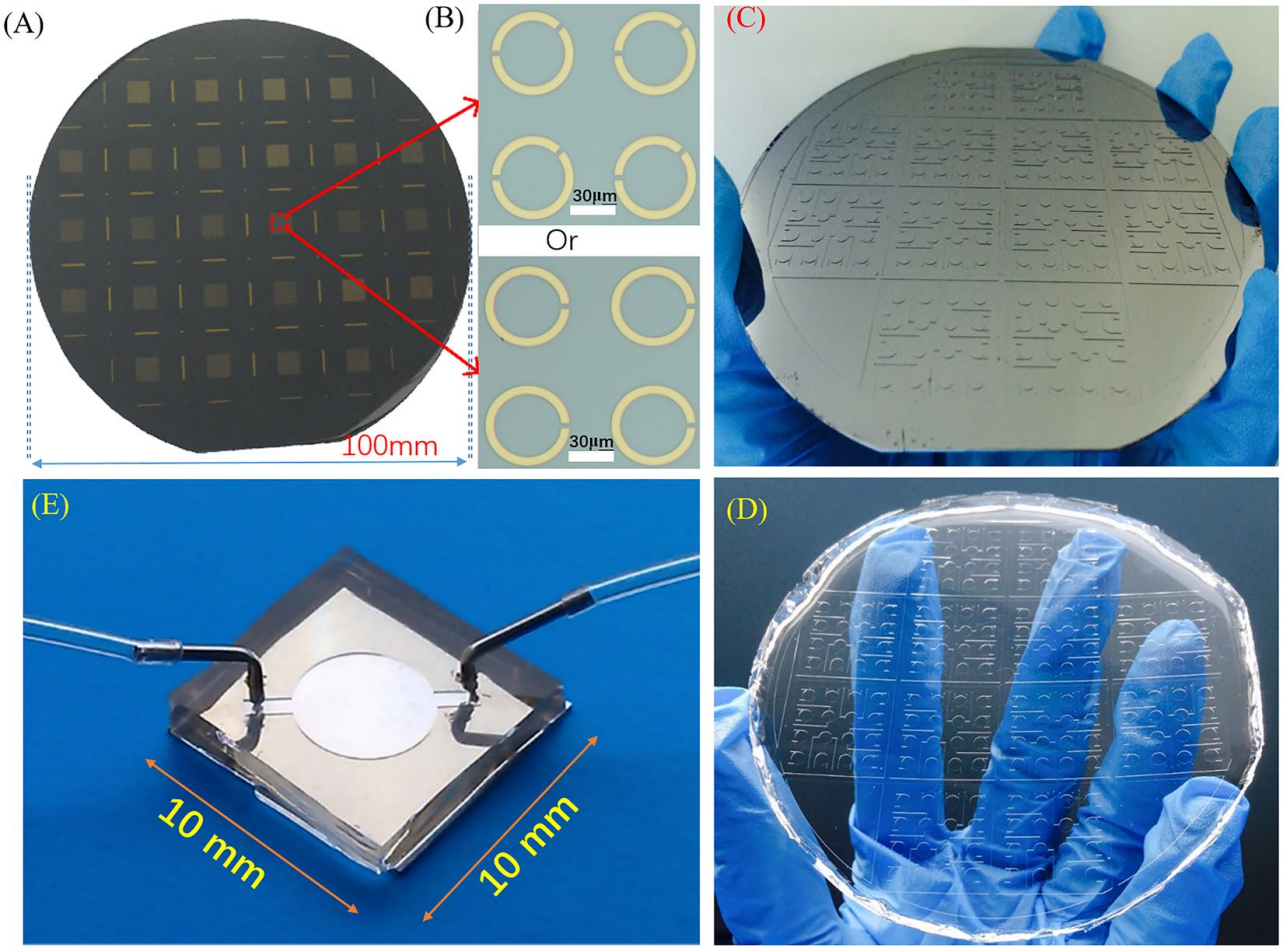
Fabrication results of SRRs and PDMS microfluidics. (**A**) Metamaterials structures on 4-inch wafer; (**B**) Local four units of the metamaterials with one or two gaps; (**C**) The SU-8 mold for microfluidics chip fabrication; (**D**) PDMS microchannel; (**E**) The THz metamaterials biosensor chip integrated with microfluidics after package.

The designed SRRs structure was fabricated on a 4-inch high resistivity silicon wafer with 1 mm thickness and more than 5000 Ω•cm by MEMS technology to get a high transmittance. The main fabrication processes included RCA standard cleaning, lithography, deposition, and lift-off (shown in Fig. 5). The detailed information of the fabrication processes was described as following. Firstly, the photoresist (AR-N4340) was coated on the silicon substrate by the spin-coating method, exposed, patterned and developed. Then, a 200-nm thickness of gold was deposited by radio frequency magnetron sputtering method. Finally, the metallic layer on the photoresist was removed by the lift-off technology. The wafer was sawed to 1 cm × 1 cm samples by the automatic precision dicing saw. The fabricated results of the SRRs are illustrated in Fig. 6 (A and B). Oxygen plasma treatment for ten minutes was adopted to remove organic contamination before they are functionalized.

### Microfluidic channel fabrication

To be convenient for modifying the surface of the metamaterials biosensor chip and to control the volume of the sample, the microfluidics should be applied in this chip. Polydimethylsiloxane (PDMS) with good bio-compatibility was used to fabricate microfluidic channel. The modification of sensor surface was performed by introducing the antibody and antigen solution by microchannel. After the modification process finished, PDMS microfluidic channel layer must be peel off before the THz testing, because the transmission ratio of PDMS in THz region is less than 50%. The detailed information was described as following.

The replication technology was utilized to fabricate PDMS microchannel which was the counterpart of SRRs. Microchannel mold, which were defined by photolithography, was made from SU-8.

The illustration of the fabrication process was shown in Fig. 5(D–G). Avoiding surface contamination is essential in fabrication processes. Before fabrication, PDMS and SU-8 should be prepared with different ratio.

Firstly, one layer of SU-8 with 60 μm thickness was spun onto a 4-inch polished silicon wafer after standard cleaning. Soft bake was done at 65 °C for 10 min and subsequently at 95 °C for 30 min. The patterns of the desired structures, i.e., microchannel, inlet and outlet, were simultaneously transferred into this layer via the standard photolithography process (Ma6/BA6, Karl Süss Germany). After exposure, post exposure bake (PEB) at 65 °C for 5 min and subsequently at 95 °C for 10 min, and development were carried out sequentially. After development, the wafer was rinsed with isopropyl alcohol to remove the residual SU-8 at the un-exposure area and to form right angle in the microchannel. To enhance the adhesive power between the silicon wafer and SU-8, the wafer with SU-8 mold structure was baked at 120 °C for 120 min. Before pouring PDMS on the SU-8 molds, the surface was pre-treated with the modified mold release agent to guarantee the SU-8 mold could be used repeatedly. Then the prepared liquid PDMS pre-polymer was poured onto this SU-8 mold. Lastly, after complete curing in the furnace at 80 °C for 60 min., the PDMS layer with inverse structures having been transferred from the SU-8 mold was carefully peeled from the mold substrate. The fabricated SU-8 mold and PDMS microfluidic channel were shown in Fig. 6 (C and D). The more detailed information was described in reference^16^.

### Bonding

The schematic of the bonding process was shown in Fig. 5(H). After bonding, the Au SRRs was covered by a PDMS layer to prevent Au SRRs contaminated. The detailed processes were described as below.

PDMS pieces were ultrasonically cleaned in acetone for 10 min and rinsed with ethanol. The surface of microfluidic channel was washed. Then, PDMS with microchannel was treated with Corona-triggered PDMS bonding technique for 20 seconds. The bonding of the SRRs substrate and PDMS microchannel was instantaneous and no pressure was applied to initiate the bond. A longer bonding time (at least 24 hours.) was required to achieve reversible bonding. Lastly, the stainless-steel pipe (inner diameter 0.6 mm and outer diameter 0.8 mm) with soft and transparent plastic pipe was assembled into PDMS to connect microchannel (Fig. 5(H)). The fabricated results shown in Fig. 6(E).

### Metamaterials pre-functionalization and metamaterials-antibody conjugates

**Figure 7:**
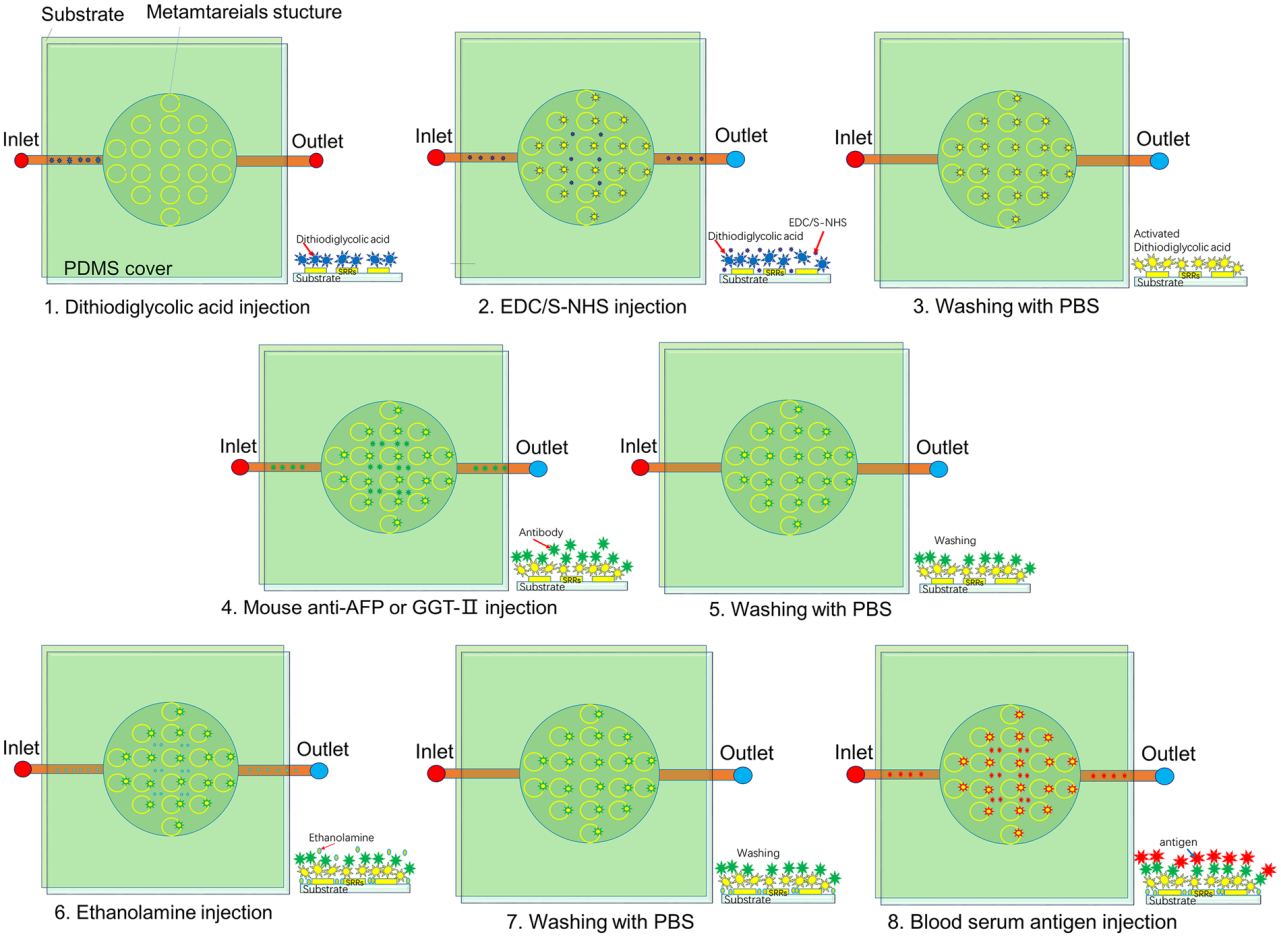
A schematic of *in situ* multi-steps (1–8) operating processes. (1) Dithiodiglycolic was injected from the inlet to cover the gold SRRS surfaces and to form a thin Au-S bonding layer; (2) The mixture of same volume EDC and S-NHS was injected in the micro-chamber and to form a thin coupling layer for protein; (3) Washing process to remove unbound EDC and S-NHS; (4) AFP antibody or GGT-II was injected in micro-chamber and is adsorbed on Au SRRS surface; (5) The redundant AFP antibody or GGT-II was thoroughly rinsed with PBS buffer; (6) Ethanolamine aqueous solution was injected, and modified the surface of the SRRS without bonding AFP antibody or GGT-II; (7) The PBS buffer was pumped in the micro-chamber to rinse out redundant ethanolamine aqueous solution; (8) The blood serum antigen was in injected in the micro-chamber.

The Au SRRs on silicon wafer was treated with following methods for functionalization. The method is almost same to the reference^16^. The protocol was also shown in Fig. 7.

Dithiodiglycolic acid (Sigma) with 2 mM aqueous solution was pumped into the chamber with low flow rate 1 μl/min. Reaction and adsorption happened in 30 min on the surface of SRRs, because the carboxyl groups on dithiodiglycolic acid were activated in 30 minutes. Redundant dithiodiglycolic acid was pumped out by 0.01 M PBS buffer with low flow rate 30 minutes later. The same volume of 1-ethyl-3-(3-dimethylaminopropyl) (EDC) with concentration 0.4 M and sulfo-nhydroxysuccinimide (SNHS) with concentration 0.1 M were injected into the chamber. The samples were thoroughly rinsed with 0.01 M PBS buffer (Sigma).

### Status before testing

**Figure 8:**
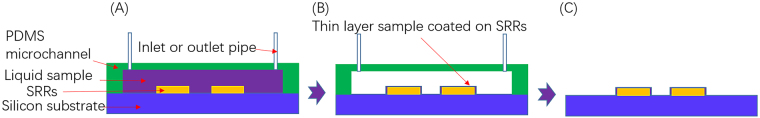
Illustration of status for THz biosensor chip before testing. (**A**) The tested sample filled chamber fully after antigen injecting chamber; (**B**) The redundant liquid sample was pumped out and N_2_ gun blow the chamber; (**C**) The PDMS microchannel was peeled off.

After *in situ* multi-steps (1–8) operating processes (in Fig. 7), the tested sample was coated on the SRRs. Meanwhile, the liquid sample was filled the whole chamber. This status S1 shown in Fig. 8A. The redundant liquid sample was pumped out (status S2, Fig. 8(B)). And then, N_2_ gun was used to blow the chamber gently and keep the surface without redundant liquid. Before mounting on THz testing setup. The PDMS microchannel was peeled off (status S3, Fig. 8(C)). Because the PDMS layer would affect the transmission of THz signal.
